# Synchrony in Joint Action Is Directed by Each Participant’s Motor Control System

**DOI:** 10.3389/fpsyg.2017.00531

**Published:** 2017-04-10

**Authors:** Lior Noy, Netta Weiser, Jason Friedman

**Affiliations:** ^1^Department of Molecular Cell Biology, Weizmann Institute of ScienceRehovot, Israel; ^2^The Theatre Lab, Weizmann Institute of ScienceRehovot, Israel; ^3^Sagol School of Neuroscience, Tel Aviv UniversityTel Aviv, Israel; ^4^Department of Physical Therapy, Sackler Faculty of Medicine, Tel Aviv UniversityTel Aviv, Israel

**Keywords:** visuomotor tracking, mirror game, intermittent control, joint action, motor control

## Abstract

In this work, we ask how the probability of achieving synchrony in joint action is affected by the choice of motion parameters of each individual. We use the mirror game paradigm to study how changes in leader’s motion parameters, specifically frequency and peak velocity, affect the probability of entering the state of co-confidence (CC) motion: a dyadic state of synchronized, smooth and co-predictive motions. In order to systematically study this question, we used a one-person version of the mirror game, where the participant mirrored piece-wise rhythmic movements produced by a computer on a graphics tablet. We systematically varied the frequency and peak velocity of the movements to determine how these parameters affect the likelihood of synchronized joint action. To assess synchrony in the mirror game we used the previously developed marker of co-confident (CC) motions: smooth, jitter-less and synchronized motions indicative of co-predicative control. We found that when mirroring movements with low frequencies (i.e., long duration movements), the participants never showed CC, and as the frequency of the stimuli increased, the probability of observing CC also increased. This finding is discussed in the framework of motor control studies showing an upper limit on the duration of smooth motion. We confirmed the relationship between motion parameters and the probability to perform CC with three sets of data of open-ended two-player mirror games. These findings demonstrate that when performing movements together, there are optimal movement frequencies to use in order to maximize the possibility of entering a state of synchronized joint action. It also shows that the ability to perform synchronized joint action is constrained by the properties of our motor control systems.

## Introduction

In order to succeed in performing a joint action, for example, lifting a heavy object together, the individual actors need to be coordinated (Sebanz et al., 2006). This social coordination can be challenging, in particular when the performed joint-action is open-ended, as in the case of jointly improvised motion (Dumas et al., 2010; Noy et al., 2011; Watanabe and Miwa, 2012; Noy, 2014; Hari et al., 2015; Gueugnon et al., 2016a; Feniger-Schaal and Lotan, 2017; Słowiński et al., 2017).

One strategy that reduces the challenge of social coordination is seeking common ground. For example, when two people are asked to independently choose a meeting point in a foreign city (e.g., Paris, the so-called Schelling game), they often manage to pick the same salient location, for example, the Eiffel Tower, from their common ground (Schelling, 1960; Clark, 1996; Vesper et al., 2011). In the context of conversation, common ground is defined as the knowledge, beliefs and assumptions of the participants about what they mutually know (Clark, 1996; Schober and Spiro, 2014). During a conversation, participants develop a hierarchy of aligned representations, the implicit common ground (Garrod and Pickering, 2004). This common ground is used to align meaning through a process of interactive alignment at lower levels such as particular choices of words or the alignment of body postures (Garrod and Pickering, 2009).

When performing joint action, people converge to an implicit common ground by moving in a more predictable way than when moving alone. For example, participants reduce the variability of their movement when they need to coordinate key presses in a reaction time task with a partner (Vesper et al., 2011) or to perform joint hopping (Vesper et al., 2013). Making your behavior more predictable is one mechanism for achieving successful joint action.

A recent finding on improvised joint motion can be interpreted according to the mechanism of convergence to an implicit common ground. In previous studies, we examined improvised joint motion using the mirror game paradigm – a theater based practice in which two actors improvise synchronized and interesting motion together (Noy et al., 2011; Noy, 2014). In the experimental one-dimensional mirror game, pairs of participants create synchronized motion together by moving handles on parallel tracks (Noy et al., 2011, 2015b; Hart et al., 2014; Zhai et al., 2015; Zhao et al., 2015; Dahan et al., 2016; Gueugnon et al., 2016a; Słowiński et al., 2017). A main finding from these mirror game studies is that players can enter a dyadic pattern of synchronous movement using predictive control. This pattern of synchronized motion is characterized by smooth and jitter-less motion, without the typical jitter resulting from reactive control in a leader-follower dynamic. This dyadic pattern was termed co-confident motion (CC motion) (Noy et al., 2011, 2015a) and has been suggested as an experimental proxy for the state of togetherness (Hart et al., 2014; Hari et al., 2015; Noy et al., 2015b), a dyadic state high synchrony and high performance, related to the notions of *group flow* (Sawyer, 2008) and *being in the zone* (Seham, 2001; Noy, 2014).

In a recent work, we analyzed the kinematic properties of basic movement elements (motion strokes between stopping events) during CC motion (Hart et al., 2014). We found that different players converge to a canonical pattern when they enter the dyadic state of CC motion. This canonical pattern consists of symmetrical basic movements, resembling a sine wave. These movements do not have the individual tendencies observed when players are in a leader-follower dynamic, for example, the tendency to move in a non-symmetric way with high skewness. It seems that during CC motion segments, participants shed their individual motion style in order to reach a common ground that supports synchronized joint action.

Interestingly, the canonical motion pattern that was observed in synchronized CC motion was identical to the optimal solution of a well-known computational motor control model. According to the minimum jerk model – a classical motor control model that describes a wide variety of human movements (Flash and Hogan, 1985) – the optimal solution for rhythmic motion (as oppose to point-to-point motion) is a sine wave (Hogan and Sternad, 2007). It is possible that during CC periods, the two players converge to a canonical pattern stemming from an optimal state of each participant’s motor control system. This connection suggests a general mechanism for achieving synchrony in joint action: finding the common ground stemming from the *similar* motor control systems of the two participants.

To test this idea, we looked for a feature of participants’ motor control systems that will direct the choice of motion parameters during synchronized joint action to a specific ‘sweet spot.’ One clue was an auxiliary finding in Hart et al. (2014). In the supporting information, we analyzed the peak velocity and frequency of motion segments within and outside CC motion. We found that CC segments tend to occupy a different region of the velocity-frequency space to leader segments. In particular, CC motions tend to have shorter durations, with motion frequencies in the range of 0.6 – 1 Hz (see Supplementary Figure S5 in Hart et al., 2014). It seems that the ‘sweet spot’ for achieving synchronization in the mirror game is for movements at relatively high frequencies.

Several studies from the field of motor control suggest a mechanism that explains this preference for achieving synchronization at higher frequencies. It turns out that that people cannot perform smooth motions (i.e., with a single peak in the velocity profile, or equivalently, without jitter) that are longer than a certain duration (Morasso et al., 1983; Milner, 1992; Vikne et al., 2013). In the context of motor control, a smooth motion without jitter is often considered as a submovement, a central concept in the theory of intermittent control (Navas and Stark, 1968; Miall et al., 1986, 1993; Burdet and Milner, 1998; Morasso et al., 2010). According to this theory, for point-to-point movements with a longer duration than a certain threshold (that is, below a certain frequency of motion) the motor control system cannot produce a single smooth motion (with a single peak in the velocity profile) but rather divides the motion into multiple, overlapping submovements, which results in jitter and non-smooth motion. For example, van der Wel et al. (2009) showed that people can produce smooth motions only up to a duration of approximately 1000 ms (corresponding to a frequency of 0.5 Hz).

To summarize, we hypothesize that participants cannot perform CC using relatively long duration motions (low frequencies), as these motions cannot be performed with a single velocity peak due to limitations of the motor control system. Supporting this hypothesis is a recent finding from our lab where we analyzed the motor control mechanisms underlying the mirror game using controlled perceptual-manual tracking tasks (Noy et al., 2015a). In that work, we found that the rate of participants’ jitter motion increases at lower frequencies of the tracked stimuli (see Figure 4B in Noy et al., 2015a). As CC motion requires no jitter, this finding supports the notion that participants will perform more CC motion as the frequency of the tracked stimuli is higher.

To test this hypothesis we followed a dual-track route, analyzing both tracking experiments using fixed stimuli, and the more ecological dyadic mirror games. The mirror game is an open-ended task and hence is challenging for testing specific hypotheses, as the experimenters do not have control over the range and variation of performed motion. To overcome this, we previously suggested supplementing the mirror game with controlled experiments focusing on the perceptual-manual tracking facet of the game (Miall et al., 1986, 1993; Noy et al., 2015a). Here, we follow this route by asking participants to manually track continuous one-dimensional movements that were displayed on a computer screen, in a setup similar to the experimental mirror game (Hogan and Sternad, 2007; Elliott et al., 2009; Degallier and Ijspeert, 2010). This enables us to create an evenly designed set of stimuli with different combinations of frequencies and velocities. The same set of stimuli was presented to all participants, and we hypothesized that the probability of CC motion (synchronized and smooth motions produced by the participants in the manual tracking) should increase as a function of the frequency of the presented stimuli.

In addition, to connect our findings to the field of joint action and social coordination, we performed the same analysis on a series of datasets from two-player experimental mirror games collected in previous studies (Noy et al., 2011, 2015b; Hart et al., 2014; Feniger-Schaal et al., 2016). These datasets include pairs of expert improvisers, and pairs of a repeated expert and a novice, in different conditions (e.g., round duration, leader/follower role). The current study therefore studies the effect of stimuli frequency on the rate of CC motion both in a well-controlled single person tracking task, and in a more ecological and open-ended two-person task.

## Materials and Methods

### Participants

Eighteen right-handed participants participated in the experiment, from the student population at Tel Aviv University (age 21–29, 12 females). Right handedness was confirmed using the Edinburgh inventory (Oldfield, 1971). The study was approved and carried out in accordance with the Tel Aviv University Human Ethics committee, and all participants gave written informed consent in accordance with the Declaration of Helsinki. The participants were paid for their participation.

### Apparatus

Data was collected using a digital graphics tablet (30.5 cm × 45.5 cm, Intuos2, Wacom Ltd), with a Samsung computer monitor (29.5 cm × 53.3 cm) used to display feedback of the hand position in the various conditions. Data collection was carried out using the RepeatedMeasures software (Friedman, 2014), and the data was analyzed using custom Matlab (MathWorks, Inc.) scripts.

### Experiment Setup

**FIGURE 1:**
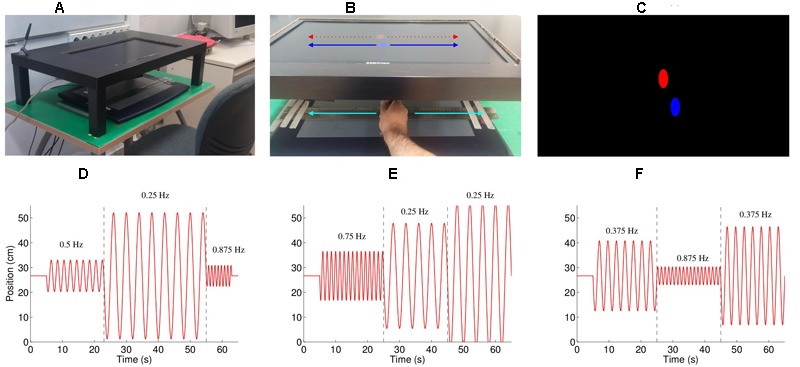
**Experimental setup. (A)** The experimental setup consisted of a Wacom Intuos 2 tablet situated under a table, such that the participant could not see their moving hand. The participants moved the stylus left and right within a channel formed by two metal rulers. **(B)** Feedback on the position of the stylus was provided by a blue oval, which moved left and right exactly the same amount as the hand moved left and right. The participants were instructed to follow the movement of the red oval, which also moved only left-right. **(C)** A screenshot of the experiment, showing the red, computer controlled oval, and the blue, participant controlled oval **(D–F)**. Three example of the stimulus (trials 2, 3, and 7), consisting of concatenated half-sine waves. The numbers on the graphs indicate the frequency of that part of the movement (separated by the dashed lines). The peak velocities for the three segments were **(D**: trial 2) 20.0, 40.0, and 26.7 cm/s; **(E**: trial 3) 46.6, 33.3, and 46.6 cm/s; and **(F**: trial 7) 33.3, 20.0, and 46.6 cm/s.

The participant was seated in front of a table, on which the graphics tablet rested (see **Figure 1A**). A custom-made shelf (made by cutting a hole in the top of an IKEA^TM^ LACK coffee table and trimming the legs) was placed directly above the tablet, which held the computer monitor that displayed feedback such that it was positioned 20 cm above the tablet. The seat height was adjusted so that the participant could move their hand freely on the tablet. The participant held a stylus in their dominant (right) hand; movements were restricted to 1D (left–right movements) by creating a track with two metal rulers. The location of the tablet and the screen was calibrated such that the location of the feedback shown on the screen was exactly above the actual position of the stylus (under the stand), participants could only see this feedback presented on the screen, and not their hand movements directly. We estimated the delay between movement of the hand and visual feedback of its location at approximately 80 ms, using a high-speed camera (120 Hz) camera, by comparing in a test the first frame when the hand moves compared to the first frame when the ellipse moves. This is comparable to the values found in similar setups (Zopf et al., 2015). This delay was not noticeable to the subjects, particularly as subjects could not see their hands moving, only the feedback.

### Experimental Protocol

An oscillating stimulus, consisting of half-sine waves, was shown as a red ellipse moving horizontally (**Figure 1B**), with each trial beginning with the red ellipse appearing in the center of the screen followed by a gong sound. As the stylus touched the tablet a blue ellipse appeared above its location (**Figure 1C**). The participants were instructed to imitate the movement of the red ellipse with the blue ellipse by moving the stylus left and right. The task included 11 one-minute trials, with breaks between each trial. The frequencies of the movements were selected from the frequencies 0.25, 0.375, 0.5, 0.625, 0.75, and 0.875 Hz, and the peak velocities selected from 20.0, 26.7, 33.3, 40.0, and 46.6 cm/s, such that each frequency and peak velocity occurred approximately the same number of times. Each trial consisted of three < frequency, peak velocity > combinations (e.g., see **Figures 1D–F**), apart from trials 1 and 6 which consisted of only two combinations. The complete set of stimuli is described in **Table 1**, and is available for download (Noy et al., 2016). To prevent discontinuities in the velocity profiles, we replaced the position and velocity between 250 ms before to 250 ms after the join (points where the prescribed frequency and/or amplitude change) with a third order polynomial fit to match the position and velocity at its start and end, thus ensuring that the position and velocity were continuous throughout the trial. The order of the trials was randomized for each participant.

**Table 1:** Stimulus properties.

Stimulus number	First-third	Second-third	Final-third
1	0.25 Hz, 40.0 cm/s		0.5 Hz, 26.7 cm/s
2	0.5 Hz, 20.0 cm/s	0.25 Hz, 40.0 cm/s	0.875 Hz, 26.7 cm/s
3	0.75 Hz, 46.6 cm/s	0.25 Hz, 33.3 cm/s	0.25 Hz, 46.6 cm/s
4	0.25 Hz, 26.7 cm/s	0.875 Hz, 40.0 cm/s	0.75 Hz, 33.3 cm/s
5	0.625 Hz, 46.6 cm/s	0.75 Hz, 26.7 cm/s	0.375 Hz, 40.0 cm/s
6	0.375 Hz, 20.0 cm/s		0.625 Hz, 40.0 cm/s
7	0.375 Hz, 33.3 cm/s	0.875 Hz, 20.0 cm/s	0.375 Hz, 46.6 cm/s
8	0.875 Hz, 46.6 cm/s	0.75 Hz, 20.0 cm/s	0.625 Hz, 26.7 cm/s
9	0.5 Hz, 33.3 cm/s	0.625 Hz, 33.3 cm/s	0.25 Hz, 20.0 cm/s
10	0.375 Hz, 26.7 cm/s	0.875 Hz, 33.3 cm/s	0.5 Hz, 46.6 cm/s
11	0.75 Hz, 40.0 cm/s	0.5 Hz, 33.3 cm/s	0.625 Hz, 20.0 cm/s

*The stimuli consisted of repeated half-sine waves, which started and ended at the horizontal midline of the screen, and alternated between movements to the left and right. Each trial consisted of three combinations of frequencies and peak velocities, with the exception of trials 1 and 6, where a single frequency/peak velocity combination was shown for two thirds of the trial. Due to different durations of the half-sine waves, each frequency/peak velocity combination was not exactly the same length, rather they were selected to be approximately one third of the trial duration (i.e., 20 s).*

### Data Analysis

**FIGURE 2:**
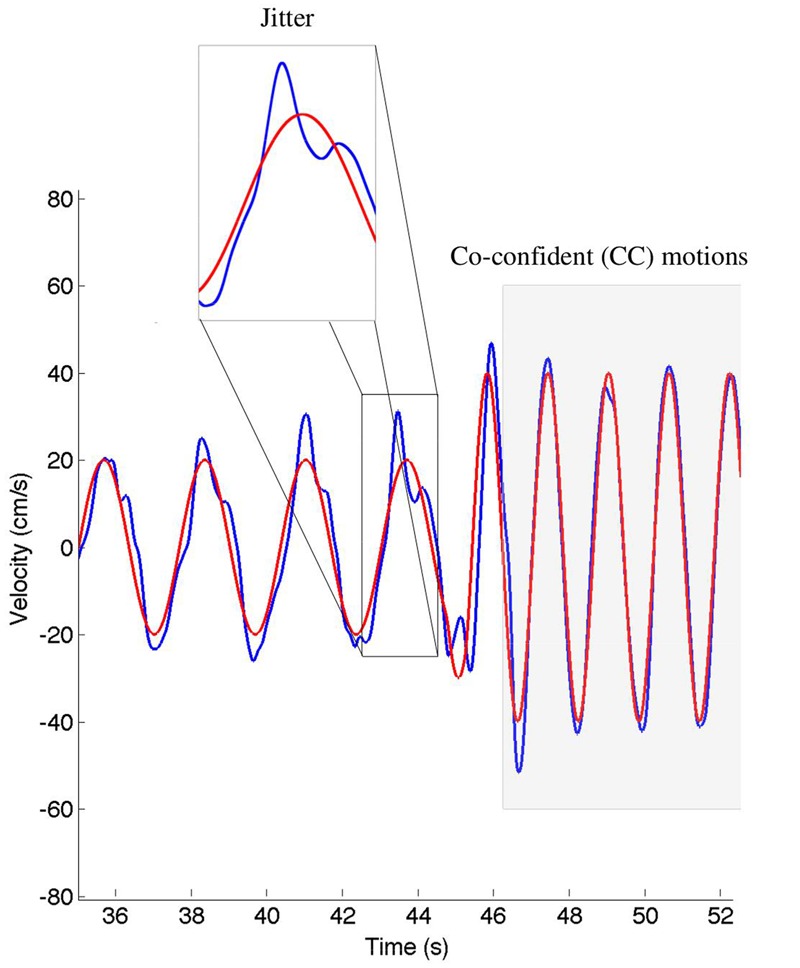
**An example of the classification of the co-confident (CC) motion segments, which are highlighted in gray.** During these regions, the movement is synchronized and without jitter. This can be contrasted to the cutout, where significant jitter can be observed. The stimuli are shown in red, the response in blue.

We calculated the relative position error (dX), relative velocity error (dV), and mean timing error (dT) using the techniques described in Noy et al. (2015a). These values are reported in **Table 2**. The jitter and co-confident (CC) periods were computed using the same techniques described previously (Noy et al., 2015a,b). Briefly, we found the best registration of the data with the stimulus (Tang and Müller, 2008). We determined the locations of acceleration zero crossings (AZC), and removed those that corresponded to AZC in the stimuli. The remaining AZCs were defined as the jitter points. The jitter frequency is calculated as half the reciprocal of the distance between jitter points. Segments of movements were classified as CC if they contained exactly one AZC (i.e., no jitter), and the stimuli and response were fairly similar [dV < 0.95, dT < 0.15 s; see Noy et al. (2015a) for definitions of these measures]. **Figure 2** shows examples of jitter and CC regions. Values are presented as means ± standard deviation. 95% confidence intervals are presented for all parameter estimates.

**Table 2:** The values shown are the mean and standard error over the 18 participants.

Stimulus number	Relative position error (dX)	Relative velocity error (dV)	Mean timing error (s) (dT)	Peak jitter frequency (Hz)	%CC
1	0.37 (±0.04)	0.72 (±0.03)	0.09 (±0.00)	0.60 (±0.03)	36.67 (±4.64)
2	0.34 (±0.02)	0.92 (±0.03)	0.08 (±0.00)	0.64 (±0.03)	52.90 (±4.76)
3	0.60 (±0.05)	1.02 (±0.06)	0.07 (±0.01)	0.68 (±0.04)	27.71 (±4.85)
4	0.41 (±0.02)	0.81 (±0.04)	0.06 (±0.00)	0.57 (±0.04)	48.30 (±5.10)
5	0.39 (±0.04)	0.89 (±0.06)	0.06 (±0.00)	0.47 (±0.03)	36.55 (±3.87)
6	0.43 (±0.02)	0.71 (±0.03)	0.06 (±0.00)	0.58 (±0.05)	39.24 (±4.69)
7	0.42 (±0.05)	0.99 (±0.06)	0.07 (±0.01)	0.50 (±0.03)	37.21 (±4.50)
8	0.24 (±0.02)	0.93 (±0.05)	0.06 (±0.00)	0.37 (±0.03)	39.15 (±4.80)
9	0.42 (±0.01)	0.89 (±0.03)	0.07 (±0.01)	0.66 (±0.03)	25.55 (±3.78)
10	0.43 (±0.02)	0.98 (±0.04)	0.07 (±0.00)	0.51 (±0.03)	44.00 (±5.28)
11	0.39 (±0.02)	0.84 (±0.05)	0.06 (±0.00)	0.39 (±0.03)	43.39 (±4.72)

*Relative position error (dX) and relative velocity error (dV) are unitless.*

#### Similarity between Participants’ CC Segments

We measured the similarity between participants’ CC segments. We first separated the 11 trials to sections of fixed stimuli (a specific pair of frequency and peak velocity). This resulted in 31 segments (from nine trials with three sections and two trials with two sections, see **Table 1**). We converted the motion traces in each section (position vectors) from all participants to CC vectors with the same length, containing 1 for time points that were inside motion segments that were detected by the automatic CC algorithm (CC segments) and 0 otherwise. We next compared for each trial section, all possible pairs of CC vectors from different participants (yielding 153 comparisons, from our *N* = 18 participants). We computed the Hamming distance for each comparison of two CC vectors (coming from different players responding to the same stimuli). We averaged the Hamming distance of each pair of players over the 153 pairs to arrive at a distance score (between 0 and 1). The resulting 31 distance scores reflect the average distance between CC responses for a given stimuli (trial section).

To test the statistical significant of these distance scores, we compared them to distance scores of shuffled data. To create a single shuffled dataset, we repeated the above procedure with one difference. When stacking together CC vectors of our 18 participants we randomly chose for each participant a CC vector that is, a CC vector from the same participant but from any of the 11 trials. Notice that we did not shuffle the order of the section, that is, the shuffled data compared the response of players to the same section (first, second, or third) in different trials. For a single shuffled dataset, this procedure resulted in one set of 31 simulated distance scores similar to the real distance scores. We repeated this procedure 10,000 times, and averaged across all simulations to get a set of simulated distance scores from the shuffled data. We then computed the statistical difference between the real distance scores and the simulated distance scores using a matched-pair *t*-test.

#### Dependence of CC Probability on Frequency and Peak Velocity

We plotted a histogram of CC probability as a function of stimulus frequency, using the CC values described above. The stimuli frequency could only take one of six values, due to the experimental design. We similarly plotted the CC probability as a function of the peak velocity (one of five values).

#### CC Probability in Two-player Mirror Games

We computed the CC probability in two-player games, taken from previous studies, as a function of motion frequency. These data sets were collected in previous studies on the two-player mirror game (Noy et al., 2011; Hart et al., 2014; Feniger-Schaal et al., 2016). We looked at three data sets: “Expert–Expert (EE),” “Novice-Expert 1 (NE1),” and “Novice-Expert 2 (NE2).” Description of the three data sets appears in **Table 3**. Note that in contrast to this study, the frequency of the motion can take any value. To allow easy comparison with the current study, we used the frequencies selected in this study as the bin centers in the histogram, which means that the number of entries in each bin will differ.

**Table 3:** Details of the data used to calculate CC proportion from two-player games from previous studied.

Data set	Participants	Number of games	Number of rounds	Duration of rounds	Leadership in rounds [Red (R), Blue (B), Joint (J)]	Source
EE	Nine pairs of expert improvisers	9	10	Nine 1 min rounds + final 3 min round	#1..9: RBJBJRJBR #10: J	Noy et al., 2011
NE1	Two repeating (male and female) expert improvisers, playing with 16 male novices and 8 female novices (gender matched games)	24	3	3 min	[novice = Blue, expert = Red] BRJ	Hart et al., 2014; Feniger-Schaal et al., 2016
NE2	One repeating female expert improviser, playing with 31 male novices and 8 female novices	39	3	3 min	[same] BRJ	Unpublished data

#### Comparison of CC Probability across Different Experiments

We compared the CC probability in the different experiments using a mixed-design ANOVA, with between-subjects factor of experiment [four experiments – experiment from this paper (TP), and the three two-player games: EE, NE1, and NE2], and a within-subject factor of frequency (six values). Tukey’s honest significant difference test was used for *post hoc* comparisons.

## Results

### Participants Succeeded to Track Mirror-Game Like Motion

As expected, the participants could successfully track the stimuli, with relatively little error. The tracking errors are shown in **Table 2**, which can be compared to Table 2 from Noy et al. (2015a), from where it can be observed that the errors are of a similar order of magnitude. It should be noted, however, that in the Noy et al. (2015a), study, the stimuli were unpredictable, whereas in this study they were largely predictable. This may explain why in this study we found lower dX and mean timing errors (dV), as well as lower jitter frequency rates and much higher %CC values.

### CC Segments Are Similar across Participants

**FIGURE 3:**
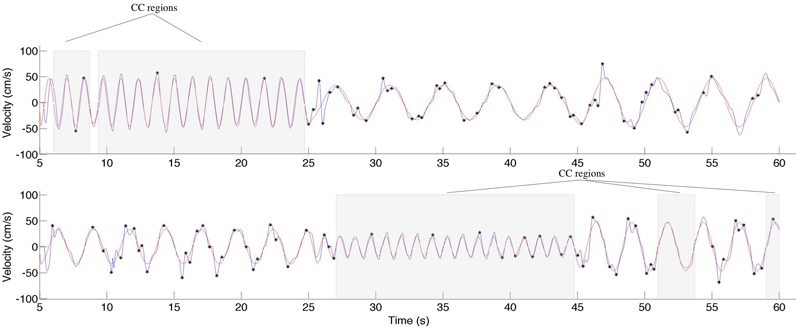
**Two examples of stimuli and responses from participant 15, trial 3, and participant 11, trial 7.** The stimuli are shown in red, the response in blue. The black stars indicate the observed jitter points (acceleration zero-crossings, not due to the stimuli), and the gray background indicates regions of CC. Note that CC is only observed for the relatively high frequencies.

**FIGURE 4:**
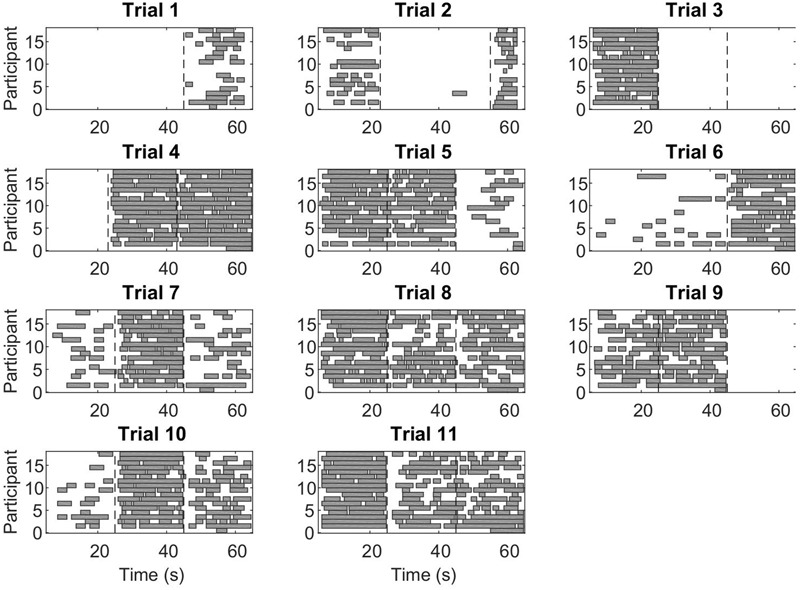
**Co-confident (CC) periods.** Each rectangle shows a continuous CC period for an individual participant, in the given trial. The vertical dashed lines indicate a change of stimulus (frequency and/or peak velocity). Note that while for some stimuli combinations, there is no CC observed at all, for other stimuli combinations, nearly all participants show CC.

During CC segments, the participants move in synchrony with the stimuli, and show little or no corrective jitter movements. Two examples of stimulus and response are shown in **Figure 3**. In the CC segments, shown in gray, there is almost no jitter corrections (i.e., AZCs, shown as black stars), and the participant’s velocity profile is very close to the velocity of the stimulus. Different trials showed different amount of CC motion (see **Table 2**, last column), because of the different stimulus properties. The CC segments for all stimuli and participants are shown in **Figure 4**, with the dotted lines indicating the time of the change in frequency and/or peak velocity of the stimuli (different trial sections). It can be observed that there is much overlap between participants in their CC regions.

To test this, we computed the distance score of CC vectors of different participants in each trial section (see Materials and Methods), and compared it to simulated data (see Materials and Methods). As expected, the distance scores from the real data (mean ± SD: 0.27 ± 0.16) was lower than the average distance scores from the simulated shuffled data (0.47 ± 0.03), and these differences were statistically significant (matched paired *t*-test: *t*(10) = -6.67, *p* < 0.001, 95% CI = [0.22 -0.33]).

### Probability of CC Is Predicted by the Frequency of the Stimuli

**FIGURE 5:**
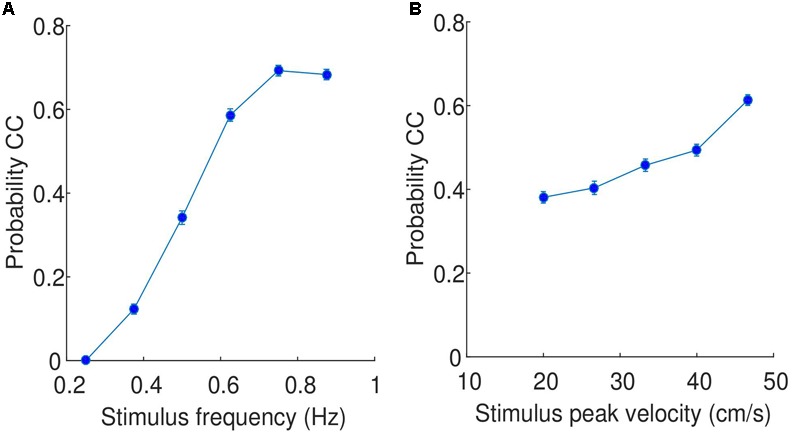
**Relationship between (A)** stimulus frequency and probability of CC, and **(B)** stimulus peak velocity and probability of CC. The data is pooled across all participants and trials. The values on the x axis are the selected stimulus frequencies/peak velocities, while the error bars indicate the standard error.

In the previous section, it was shown that CC segments are relatively consistent across participants, which implies that the probability of observing CC is a function of stimulus properties. Using data binned for all participants and trials, we showed that the probability of CC is a function of the frequency of the stimuli (see **Figure 5A**), specifically the probability of observing CC increases dramatically as a function of stimulus frequency, with no CC observed for any participant at the lowest frequency stimuli used in this experiment (0.25 Hz). As the stimulus frequency increases, the probability of observing CC increases. To test whether this result is significant, we performed the same comparison but individually for each participant. We then tested whether the slope of the regressions lines was significantly greater than zero, and found that for all participants, the slope was indeed greater than zero, this difference is supported by a *t*-test (*t*(17) = 24.48, *p* < 0.0001, 95% CI = [1.09 1.30]). In the Supplementary Material, we show that this finding is not simply a result of the CC detection algorithm used.

A similar comparison can be performed with peak velocity, shown in **Figure 5B**. While the probability of observing CC does increase as a function of increasing peak velocity, the change of probability is much less dramatic (approximately from 0.4 to 0.6). This increase is observed consistently across participants, with all participants showing slopes of regression lines greater than zero, supported by a *t*-test (*t*(17) = 11.72, *p* < 0.0001, 95% CI = [0.007 0.009]).

### Relationship between Movement Frequency and CC Is Also Found in Two-player Mirror Games

In the previous section, we showed that the probability of CC can be predicted by the frequency of the tracked stimuli, for a one-player version of the mirror game with largely predictable stimuli shown on a computer screen. In contrast, in the regular two-player version of the mirror game, the motions (movements of a handle) are chosen in an open-ended manner by the players. To test whether the effect of stimuli frequency on the probability of achieving CC generalizes to this version of the mirror game, we performed a similar analysis with data from three additional data sets, described in **Table 3**.

**FIGURE 6:**
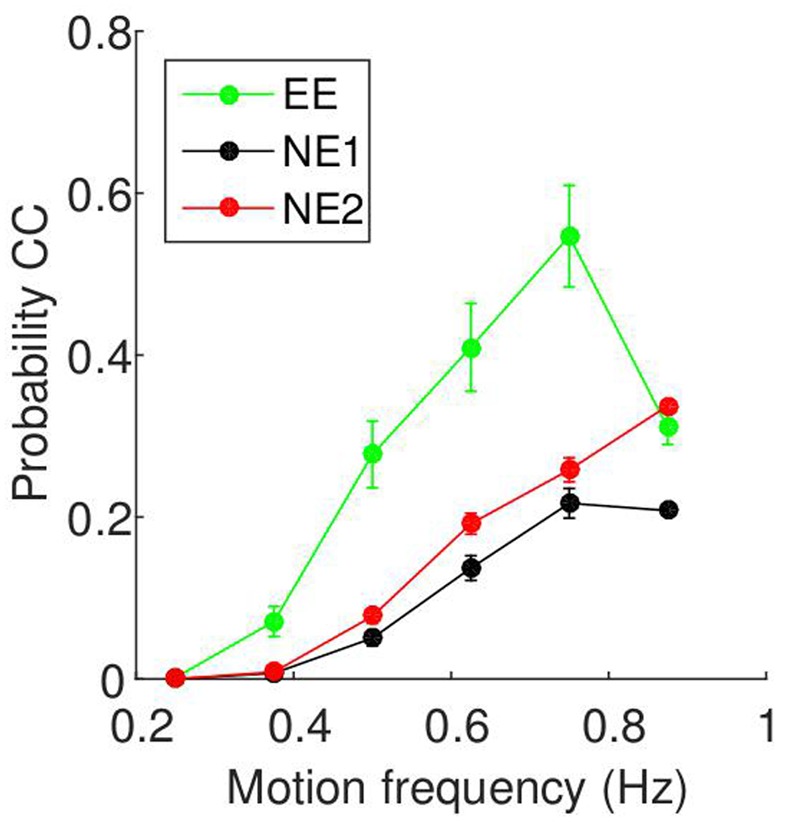
**Relationship between motion frequency and probability of CC taken from two-player mirror games.** The data is as presented in **Figure 5**, but the data is from two-player mirror games run in previous experiments. Details of the three groups (EE, NE1, and NE2) can be found in **Table 3**.

The relationship between motion frequency and CC probability are shown in **Figure 6**. For all three experiments, the probability of CC is zero at low motion frequencies, and increases as the motion frequency increases. Unlike the results from the current study, there is a drop-off at a higher motion frequency. To determine whether this result is seen across subjects, we again fitted a regression line for each participant, and tested whether they are positive using *t*-tests. For all three groups, we found positive slopes for all subjects, supported by *t*-tests (EE: *t*(8) = 4.04, *p* = 0.004, 95% CI = [0.27 1.00]; NE1: *t*(23) = 9.76, *p* < 0.0001, 95% CI = [0.32 0.49]; NE2: *t*(38) = 12.45, *p* < 0.0001, 95% CI = [0.54 0.74]).

### Comparison between the Experiments

We compared the four experiments using a mixed-design ANOVA. The CC probability differed between the groups, as shown by a main effect of experiment [*F*(3,85) = 42.9, *p* < 0.001]. Pairwise comparisons revealed that the percentage of CC in the experiment described in this paper (TP: 39.1 ± 2.1%) was significantly higher than those in the other three groups (EE: 22.5 ± 3.1%; NE1: 10.1 ± 1.8%; NE2: 15.3 ± 1.4%; *p* < 0.001 for all three). Additionally, the EE group show significantly higher CC probabilities than the NE1 group (*p* = 0.005), but the NE1 groups was not significantly different from the NE2 group. There was also a main effect of frequency [*F*(5,425) = 168.4, *p* < 0.001], with each subsequent frequency showing a CC probability significantly higher than the previous frequency (*p* < 0.001), apart from the last pair (0.75 and 0.85 Hz), which were not significantly different (*p* = 0.326). Finally, there was an interaction of experiment and frequency [*F*(15,425) = 13.85, *p* < 0.001], which demonstrates that the slopes were different for each experiment. In particular, while the differences are very small for low frequency stimuli (0.25 Hz), with the differences between groups ranging from 0% (TP and NE1/NE2; not significant) to 1.9 ± 0.7% (TP and EE; *p* = 0.04), for the higher frequencies, there is a greater difference between the groups. For example, at 0.875 Hz, the differences range between 7.3 ± 6.5% (TP and EE; not significant) and 32.3% (TP and NE1; *p* < 0.001).

## Discussion

We analyzed participants’ ability to manually track piecewise constant stimuli, simulating the behavior of a follower in a mirror game. The ‘virtual leader’ produced the same movements across different participants. By using the same stimuli (which is not the case in the regular mirror game), we were able to expose reoccurring patterns in human motion synchronization behavior. In particular, we focused on participants’ co-confident (CC) motion periods, and their relationship to the tracked stimuli frequency and peak velocity.

We found that participants successfully tracked the virtual leader’s motion. The manual tracking was done with lower errors compared to Noy et al. (2015a). This difference is probably due to the fact that the stimuli in the current study were more predictable and less complex than in the previous work. CC regions were strikingly similar across participants (**Figure 4**), a fact that can be observed due to the repeated stimuli used in the current, one-player version of the mirror game.

The main finding of this work is that the probability of CC was well predicted by the frequency of the stimulus. At low frequencies (slow movements), there was no CC at all, and the amount of CC increased as the frequency increased. The effect of the magnitude of the peak velocity of the stimulus on the probability of CC was much smaller. This finding was corroborated with the analysis of three data sets from studies employing the two player mirror game. While there is an imbalance in the two experimental designs (one person vs. two people; predetermined stimuli vs. individually selected stimuli), we suggest that the similar findings strengthen our claims that this is a general principle and not specific to the types of game.

Numerous studies have examined the question of perception-action coupling (Kelso et al., 1990; Prinz, 1997; Wolpert and Ghahramani, 2000; Rizzolatti and Sinigaglia, 2010), i.e., the inter-relatedness or common coding of perception and action. Observing a movement being performed can trigger a representation of the necessary movement to be made, potentially as a result of mirror neurons in the brain (Rizzolatti and Sinigaglia, 2010). In this task, the participants need to predict the future location of the stimuli in order to succeed in producing smooth movements. This may be achieved through a process of neural simulation (Wolpert et al., 2003). In this study, we found that the participants were unable to generate smooth movements at low frequencies. Based on the action-perception framework, this may be a result of either an inability to predict such movements (as they are not part of our natural repertoire), an inability of the motor system to produce them, or a combination of the two.

Similar tasks have been studied in the past, including tracking tasks (e.g., Miall et al., 1993), tapping to an external cue (Repp, 2005; Repp and Su, 2013) and music tasks (Novembre and Keller, 2014). A wide variety of analysis techniques have been used, including comparing power spectrums (Miall et al., 1993), error magnitudes, neuroimaging, measures of synchrony to specific events such as metronome beats (Repp, 2005) and variability (Elliott et al., 2009) to name a few. In this task, as we were specifically looking at the question of which stimuli can be successfully copied in a smooth manner, we chose to focus our analysis on the CC measure.

The current findings demonstrate the usefulness of our approach of using controlled, single player mirror game studies to complement studies on two player mirror games. The mirror game is a useful paradigm that allows for a quantified analysis of synchronization in an open-ended joint action task. The usefulness of task is demonstrated by the large number of published studies that employ the mirror game since its origin as an experimental paradigm in 2011 (Hart et al., 2014; Słowiński et al., 2014; Noy et al., 2015b; Feniger-Schaal et al., 2016; Gueugnon et al., 2016a,b; Słowiński et al., 2016). However, the open-ended nature of the task makes it difficult to perform repeated and well-controlled experiments, as each game has different motion patterns. Using a single person mirror game with a virtual (and fixed) leader overcomes this challenge (Noy et al., 2015a). Other groups have taken this approach a step further by developing and testing models of following, leading and joint improvisation in the mirror game using well-controlled avatars and robots (Zhai et al., 2014; Zhao et al., 2015; Khoramshahi et al., 2016; Słowiński et al., 2016).

Our approach also integrates methods and findings from the fields of motor control and joint action, for studying the motor control layer of jointly improvised action. This integration is in line with recent works showing the interplay of joint action and motor control, for example, studies using motor control concepts such as synergies in the context of joint action (Riley et al., 2011; Romero et al., 2015). The current work contributes to this literature by highlighting the role of an individual’s motor control system in guiding and possibly limiting joint action.

The current work offers several contributions to the field of motor control. First, we add to previous findings showing an upper limit on the duration (or lower limit on the frequency) of smooth motion segments (van der Wel et al., 2009). By systematically manipulating both the frequency and the peak velocity of the stimuli, we replicated in a systemic way the strong effect of stimuli frequency (and to a much lesser effect, of peak velocity) on the possibility of moving in a smooth way. In addition, we showed this effect in a continuous repetitive tracking task, while previous works used point-to-point motion guided by a metronome. It will be interesting in the future to study the smoothness of participants’ movements in response to stimuli at different frequencies, presented either visually as in our manual tracking task, or using auditory cues, as in the metronome driven tasks of van der Wel et al. (2009).

In general it seems that human prefer not to make slow, long duration movements, although these movements may use less energy (Berret and Jean, 2016). This is likely because there is also a cost to making longer duration movements, for example attentional or metabolic costs. Shadmehr (2010) suggested *temporal discounting* as an explanation for the tendency to avoid slow movements. Temporal discounting says that given a particular movement to make, making a faster movement will lead to a larger reward; this reward can overcome the additional costs involved in making a faster movement (e.g., greater energy expenditure).

The notion of intermittent control (Navas and Stark, 1968; Miall et al., 1986, 1993; Burdet and Milner, 1998; Morasso et al., 2010; Gawthrop et al., 2011) implies that complex movements (like the movements in the current experiment) are composed of multiple submovements that are concatenated together. Each submovement is generally assumed to be smooth, for example following a minimum jerk velocity profile. Whilst the stimuli in this experiment are maximally smooth (consisting of sine waves), the participants do not generate sine waves themselves when the frequency of motion is low. Rather, they concatenate multiple submovements to approximate the shape of the sine wave, but in doing so, they produce jittery movements. In this case, as the ideal duration of the movement is fixed by the stimuli, temporal discounting cannot explain why subjects do not produce smooth and long duration submovements instead of jittery movement consisting of several submovements. The best strategy to mirror a player who uses long duration submovements is to move in a similar way, also using long duration submovements. According to the speed-accuracy trade-off (Wickelgren, 1977), these longer duration submovements should also be more accurate. Avoiding these movements – and making more intermittent corrections – leads to worse performance, and a reduction in reward. It remains an open question whether avoiding long duration submovements stems from a neural constraint, a biomechanical constraint, a lack of practice in performing such movements, or a combination of these factors.

The issue of practice raises an interesting question that can be studied experimentally. It is likely that similar to most other perceptual-manual tasks, the performance in the online tracking task of the current experiment can be improved with practice. Previous research has shown a clear distinction between the performances of experts and novices in the mirror game (e.g., Noy et al., 2011, and see also **Table 3**). The higher performance of experts in the mirror game can be the result of learning in different routes: better execution, better perception and factors related to the joint improvisation *per se* (e.g., the ability to leave a stable pattern, see Dahan et al., 2016). The current paradigm offers the opportunity to test one of these possible routes of performance improvement.

The current work also offers several contributions for the field of joint action. The mirror game is recognized as an important paradigm for joint action and social neuroscience (Hari et al., 2015) and is used as a tool for measuring and developing interventions for different social disorders (Bardy et al., 2014; Brezis et al., 2015). The analysis of CC periods is central for mirror game studies, due to its theoretical underpinning as a marker of co-predictive controllers (Noy et al., 2011; Dahan et al., 2016), and its presumed connection to the experience of ‘togetherness’ (Noy, 2014; Noy et al., 2015b). It is therefore important to understand the limits of this measure. We find that achieving CC is much easier in medium-to-fast frequency motions. During low frequency motions, there is a relatively high amount of jitter, that stems not from a dyadic failure in performing improvised joint action but from limits of the motor control systems of each individual. This is an important observation for researchers using the mirror game as an experimental and interventional paradigm.

More generally, this observation highlights the need to be extremely careful when moving from theoretical concepts (‘togetherness’) to a well-defined operational metric (CC motions). We have previously noted that the CC measure captures only a ‘thin slice’ of the phenomenon of togetherness (Noy, 2014). For example, in a previous work participants in the mirror game produced little CC at low frequencies (in line with the findings here) but sometimes reported a high level of subjective togetherness at these moments (Noy et al., 2015b). Togetherness and CC should not be treated interchangeably, and the current work further highlights this notion.

In the context of theater improvisation, the finding that motion synchronization is easier to obtain using high frequency movements is somewhat surprising. In theater improvisation the mirror game is used as an exercise for bringing actors into a state of togetherness (Noy, 2014). To enhance the chances of getting into this state of togetherness a teacher might suggest that participants should move slowly (i.e., long duration movements) and use simple and repetitive motions (Boal, 2000). In contrast, the current work shows that in the experimental one dimensional mirror game participants are better able to achieve synchronization when avoiding long duration movements.

Future studies can further analyze and explain the differences between the one dimensional and whole body mirror games. The enrichment in synchronized movements at high frequencies in the one-dimensional game vs. low frequencies in the whole body mirror game might stem from different sources. One possible explanation involves the different perceptual complexity in the two setups. In the whole body mirror game, participants freely move different body parts, including their arms, torso and legs, and their partners have to simultaneously move the same parts. In the experimental mirror game, participants perform only back and forth motions of a single end-effector. Maybe the more complex multi-part motions in the whole body mirror game cannot be tracked when movements are at high frequencies, due to increased perceptual demands. In other words, depending of the task difficulty, slowing down or accelerating the motion could be both beneficial in a synchronization task.

Another possible route can model the different costs and rewards in the two setups. In the mirror game task, participants have different costs (e.g., energy consumption, cognitive load) that are related, among other things, to the speed and the complexity of the performed motions. The relationships between these different factors can be task dependent. For example, in the one-dimensional mirror game the physical motion is constrained in a track with clear boundaries, and it is possible that cognitive or biomechanical effects reduce the costs of high-frequency motions in this setup. In a similar vein the mirror game task induces different rewards, including an inner feeling of togetherness that might be related to the state of CC motions. A future model can try to tie together these different factors. As a small step toward this goal we have recently tested the subjective experience of participants in the mirror game, and found a higher level of subjective togetherness in CC periods, reported using a continuous togetherness-dial, when participants watch a video recording of their own games (Noy et al., 2015b).

Finally, it is possible that in the whole body mirror game, participants achieve the state of togetherness with motion patterns that differ from the operational CC measure developed for the experimental mirror game. Future studies can examine these questions, and measure the kinematic patterns of synchronized motion in the whole body mirror game. It will be interesting to discover whether players similarly converge to a ‘sweet spot’ of motions when they get into synchronized motion.

Part of the inability to perform slow movements may be due to the difference between the frequencies of these movements and the resonant frequency of the body parts being moved. Limbs possess mechanical properties, which determine their resonant frequencies (Turvey et al., 1988). Making movements at close to the resonant frequency results in lower metabolic costs (Holt et al., 1995), greater stability and maximal predictability of movements (Goodman et al., 2000). The slow movements described here (0.5 Hz) are significantly slower than the resonant frequency of the muscle-limb complex of the forearm, which was observed to range from 1.1 to 2.0 Hz (Hatsopoulos and Warren, 1996), although we note that this is not a perfect model of the arm as used in this experiment. Similarly, when coordinating pendulum movements, subjects are best able to coordinate their movements when the resonant frequencies of the pendulums are similar (Schmidt and Turvey, 1994).

The main claim of the current work is that a specific limit of individuals’ motor control systems (the inability to perform long duration, smooth motions) dampens the two-person synchronization: achieving CC at low frequencies is simply not possible. There is, however, a silver lining for this limitation. As both individuals have similar bodies, which are controlled in a similar way, we can speculate that their similar motor control systems impose similar limitations on their joint action. In this sense, the similarity of the dyad’s bodies provides a common ground that supports their joint action.

This interpretation raises interesting questions about importance of similarity between actors’ motor controls and bodies in joint action. It was suggested that observers use a model of their own movement kinematics to predict the actions of others (Prinz, 1997; Sebanz et al., 2003; Colling et al., 2014). If so, a similarity of body proportions between two agents might be helpful in achieving synchronization in joint action. Previous work supported this idea by showing that people synchronize better with recording of their own actions (Flach et al., 2003; Keller et al., 2007). In the context of the mirror game, one can speculate therefore that it will be easier to perform mirroring between similar agents, for example, between two adults vs. and adult and a child. Recent studies have started to unpack these questions by showing, for example, that people with similar motion repertoires perform better together in the mirror game (Słowiński et al., 2016).

Despite the importance suggested here for the similarity of motor control systems in synchronized joint actions, it is possible that mirroring can be achieved between agents with very different bodies and motor control systems. One example is cross-species mirroring. It was shown that dolphins are able to mirror human motions by using different body configurations, for example by lifting their tail from the water in response to a sitting human lifting her leg (Herman, 2002). In other words, while we suggest here that synchrony in improvised joint action is directed by the individuals’ motor control systems, we believe that such synchrony is not totally dictated by the interacting motor control systems, and that mirroring and togetherness can be achieved via multiple routes (Rumiati and Tessari, 2002).

## Author Contributions

LN and JF conceived and designed the experiments. NW performed the data collection. LN and JF participated in the statistical analysis and interpretation of the data. LN, NW, and JF wrote the article.

## Conflict of Interest Statement

The authors declare that the research was conducted in the absence of any commercial or financial relationships that could be construed as a potential conflict of interest.
