# Supplementary material for: Synchrony in Joint Action Is Directed by Each Participant’s Motor Control System
Source: Front Psychol. 2017 Apr 10;8:531. doi: 10.3389/fpsyg.2017.00531 (PMC5385352; doi:10.3389/fpsyg.2017.00531)
Supplement: Supplementary file 1 [file Data_Sheet_1.PDF]

Supplementary Material

Synchrony in improvised joint motion is directed by each participants' motor control systems

Lior Noy, Netta Weiser, Jason Friedman*

*** Correspondence:** Jason Friedman: jason@tau.ac.il

1 Supplementary Data

To test whether the observed relationship between stimulus frequency and CC rate is simply a function of the CC detection algorithm, we generated a set of maximally smooth movements (i.e. half sine-waves) that follow the stimuli, but are perturbed in terms of their frequency and amplitude, with frequencies and amplitudes selected from a normal distribution with its mean at the stimuli value, and standard deviations of 5% and 20% of its value, for the frequency and peak velocity respectively. Figure S1 shows an example of the generated perturbed trajectories.

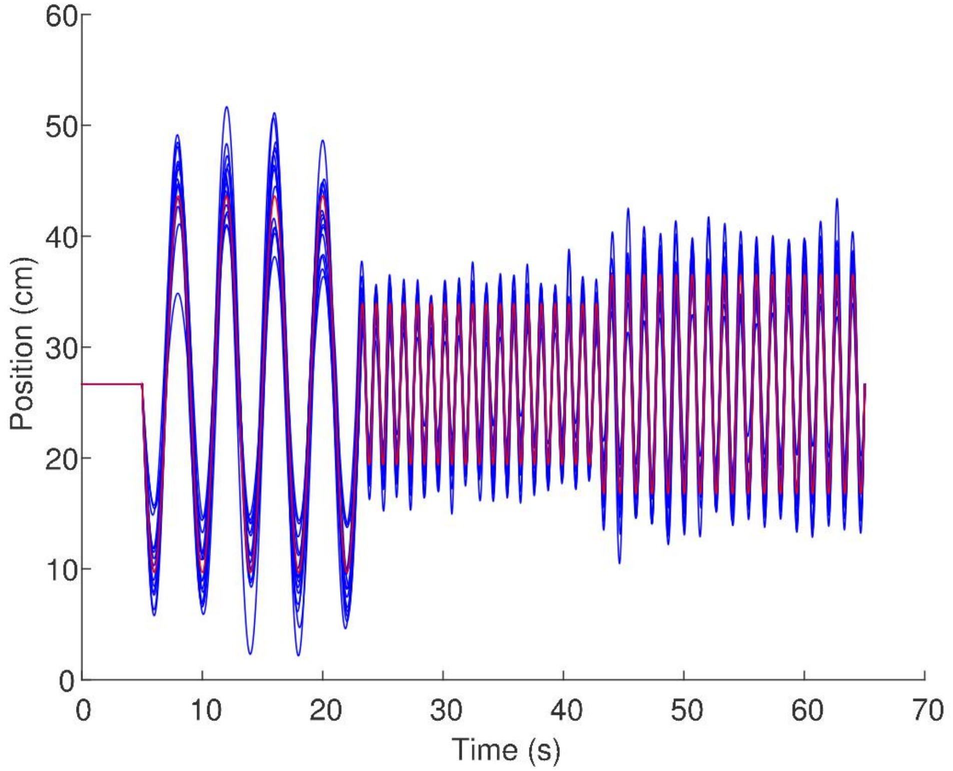

Figure S1. An example of 15 perturbed responses (for stimulus 4). The red line is the original stimulus, the blue lines are perturbed responses.

Using these generated responses, we computed the CC for each simulated trial. The results of running the simulation are shown in Figure S2.

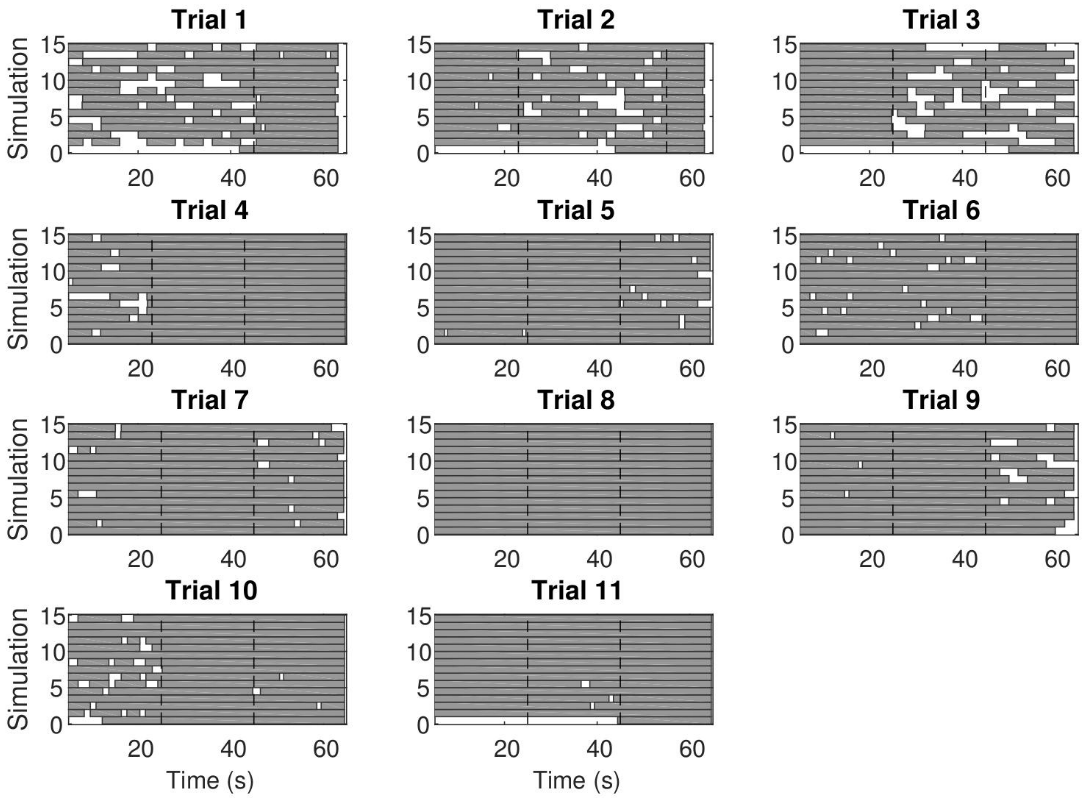

Figure S2. This figure is equivalent to Figure 4, but shows simulated rather than real data.

Overall, the rate of CC varied from approximately 70% for the lowest frequencies to 100% for the highest frequencies, as shown in Figure S3. Unlike with the subject data, CC was observed even at the lowest frequencies. Based on this analysis, we conclude that the lack of CC for low frequencies is not a result of algorithm used.

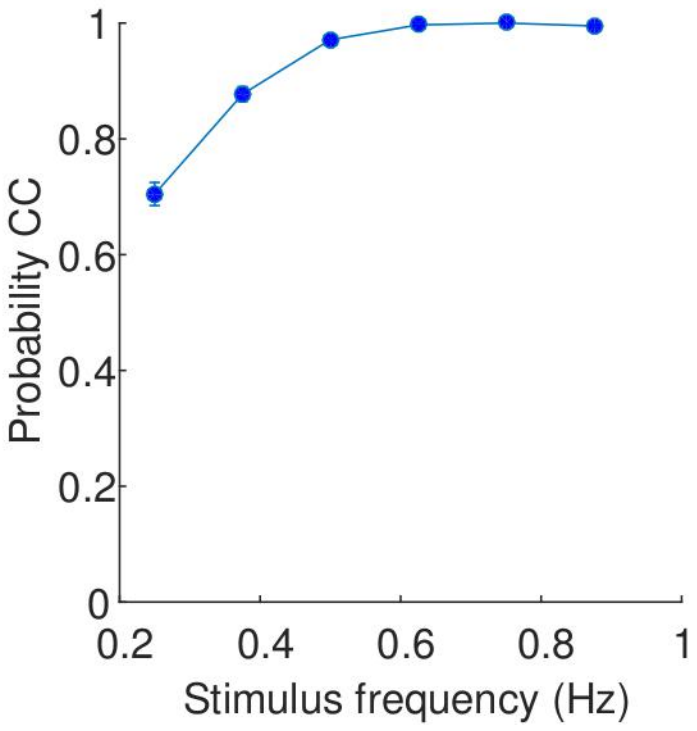

Figure S3. Relationship between stimulus frequency and probability of CC for the simulated data. Unlike for the real data, CC is observed even at the slowest frequencies.
